# Towards integrated assessments of water risks in deglaciating mountain areas: water scarcity and GLOF risk in the Peruvian Andes

**DOI:** 10.1186/s40677-020-00159-7

**Published:** 2020-09-23

**Authors:** Alina Motschmann, Christian Huggel, Randy Muñoz, Angela Thür

**Affiliations:** Department of Geography, University of Zurich, Winterthurerstrasse 190, CH-8057 Zurich, Switzerland

**Keywords:** Risk assessment, Peru, Water resources, Water scarcity, GLOF

## Abstract

Different water related risks such as lake outburst floods and water scarcity are typically assessed by separate methods and often by separate research communities. However, in a local context such as in mountain regions of the developing world different water risks are intertwined and shaped by multi-dimensional natural and socio-economic drivers. Progressing glacier melt and the associated growing number of lakes rises the threat of glacier lake outburst floods (GLOFs); at the same time declining melt water supply changes the hydrological regime, resulting in changing water availability, especially during dry seasons. Here, we address this challenge by integratively assessing water scarcity and GLOF risks and their interactions for two study sites in glacierized catchments in the Cordillera Blanca and Urubamba in the Peruvian Andes. We used hydrological modelling, GLOF flow path modelling, and interviews with local people and technical experts to assess the hazard and risks of water scarcity and GLOFs. We incorporate perspectives of people living in those areas in order to gain a more comprehensive view on risks. While metrics of flood and water scarcity hazards are difficult to compare, we found insightful results using a comparative analysis of elements at risk from different water related hazards with different probabilities of occurrence. Furthermore, our study shows that considering the diverse local perspectives on risks as well as the social, cultural, economic and political context is essential to more successful and sustainable disaster risk reduction, climate change adaptation and integrated water management.

## Introduction

In the context of climate change, risk assessments have an important role in evaluating current conditions and simulating future scenarios to assess impacts due to the changing climate. Advanced hazard and risk assessments for sudden-onset events such as debris flows, landslides or rockfall have been developed for many years and support prevention of future damage by such processes (cf. Raetzo et al. 2002; Baum et al. 2005; Hürlimann et al. 2006; Künzler et al. 2012; Frey et al. 2016). However, for slow-onset and cumulative climate change impacts, such as related to changing water resources, there is limited experience of how to apply a risk framework (Tung 2001; Lehner et al. 2006; Lei et al. 2011; Zhang et al. 2013). In fact, comprehensive analyses of risks related to water resources considering climate change within multi-dimensional drivers across different scales are complex and often missing in climate-sensitive mountain regions. Furthermore, in a multi-hazard environment where people are not only influenced by one hazard but where rather multiple hazards interplay spatially and temporarily (Kappes and Keiler 2012), hazards may need to be evaluated differently. Conceptual frameworks by international organizations, for instance the risk concept of the Intergovernmental Panel on Climate Change (IPCC), try to simplify concepts to make them broadly applicable (IPCC 2014). In the IPCC AR5, risk is defined as a function of (i) the physical climate hazard, (ii) the exposure of people, asset values, ecosystems, and (iii) their vulnerability. This concept is in principle applicable for sudden as well as slow-onset climate related events although such applications are still rare. The IPCC Special Report on extreme events and disasters (IPCC 2012) points out the relevance of adopting a multi-hazard approach in order to provide more effective climate change adaptation and risk reduction measures, both for the present and for the future.

In the highly glacierized regions of the Peruvian Andes, water resources play a fundamental role for societies and ecosystems, and glaciers can represent a crucial water resource, for local communities but also downstream urban centers, agriculture and industries (Drenkhan et al. 2015). During the dry season (austral winter) people are increasingly affected by low water availability due to shrinking glaciers supplying melt water (Bury et al. 2011; Mark et al. 2017), and unfavorable water usage and allocation (Carey et al. 2017). Rising water demands for agriculture, hydropower, domestic use and mining exacerbate problems and risks related to water availability and scarcity (low flow water risks). These risks are closely interlinked with rising water demands driven by population growth and other socioeconomic factors, such as agricultural expansion and increasing energy needs (Drenkhan et al. 2015). However, at the same time governmental risk management focuses on glacier lake outburst floods (GLOFs) as Peru is known for some of the biggest GLOF disasters and nowadays GLOFs still present a notorious threat (Carey 2010; Carey et al. 2012; Schneider et al. 2014; Emmer et al. 2016).

Vanishing glaciers, natural hazards (like inundations, mudflows, and landslides), decreasing river discharge, drying springs, next to shifts in precipitation patterns are apparent climate change impacts noticed by the local population in one way or another (Jurt 2009; Gurgiser et al. 2016; Carey et al. 2017; Heikkinen 2017; Mark et al. 2017). Nevertheless, the perceptions of people living in endangered areas differ where the risk of sudden-onset events such as GLOFs is often being underestimated due to inaccurate predictions, mitigation projects, little interaction with the hazard or believe in higher power (Dahal and Hagelman 2011; Sherry et al. 2018; Walker-Crawford et al. 2018). Research has hardly dealt with the challenge of how to integrate differing water risks in a common assessment. To consider all components of risks, i.e. hazard, exposure and vulnerability, as well as the economic and social conditions driving these components, comprehensive risk assessments are needed. Water resources management needs to deal with both high and low flow water hazards. In this context, high flow hazards refer to hazards that involve a large amount of water that exceeds the normal water flow, such as rain storms or (outburst) floods, which we will elaborate on in this study. Low flow hazards on the other hand are defined by conditions where water flow is insufficient and below normal average, such as droughts, aridity or in our case water scarcity.

Here we analyze two case studies from the Peruvian Andes where both low-flow water hazards and the high-flow hazards, i.e. GLOFs, are an issue. We assess the current and future hazards based on the impact of glacier retreat and the exposure of people and assets to both types of hazards. We complement these more technical hazard and risk assessments by analyzing different perspectives of local people in relation to those risks. Our ultimate objective is to contribute to a more comprehensive and differentiated approach to different types of water related hazards and risks which we consider important in view of climate change adaptation and disaster risk reduction. After the introduction of the case study regions we describe the methods to analyze water scarcity, GLOF hazards and the surveys and methods for gaining insights into local people’s perspectives. The results are presented separately for the two case study localities. Study sites: Chicón, Cordillera Urubamba, and Quillcay, Cordillera Blanca, Peru.

**Fig. 1:**
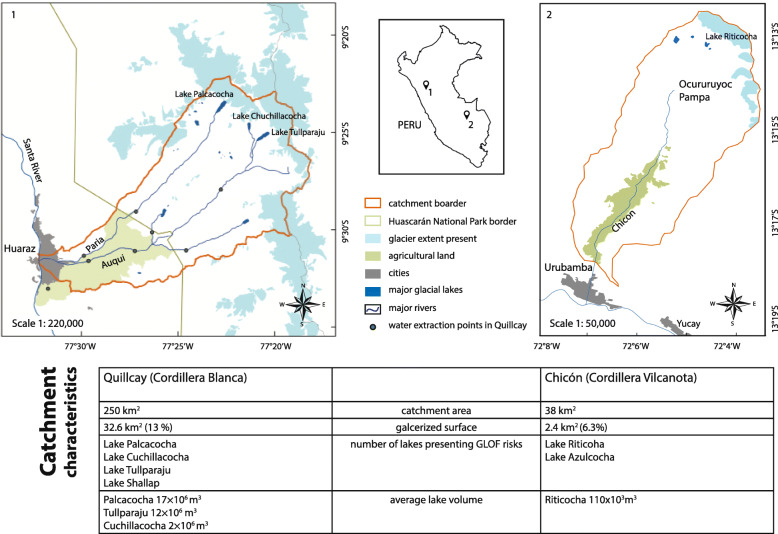
Study sites (1) Quillcay catchment in the Cordillera Blanca, (2) Chicón catchment in the Cordillera Urubamba and their catchment characteristics

Our study sites are two glaciated catchments in Peru, referred to as Quillcay and Chicón in the following (Fig. 1). Quillcay (250 km^2^) is located in the mountain range of the Cordillera Blanca in Northern Peru and Chicón (38 km^2^) in the Cordillera Urubamba, Southern Peru. Both catchments are characterized by high-altitude Andean alpine landscapes with glaciated peaks in the upper catchment (> 6000 m asl in Quillcay and > 5000 m asl in Chicón), glacial lakes that formed due to the retreat of glaciers and seasonally dry areas in the lower valleys. At the downstream end of the Quillcay catchment, the city of Huaraz (3050 m asl) is an important regional urban center (ca. 140,000 inhabitants) while in Chicón the city of Urubamba (ca. 2700 inhabitants) has a similar role as an urban center. In both sites local rural communities are scattered over the arable zones of the catchments.

Both sites experienced major GLOF events. Quillcay was hit by a GLOF in 1941 originating from Lake Palcacocha that killed approximately 1800 people (Wegner 2014) and destroyed about one third of the city of Huaraz. Chicón on the other hand was hit by a GLOF in 2010 originating from Lake Riticocha. Apart from structural damage and disruption of infrastructure in the valley and the city of Urubamba, no people were killed in the event. After the disaster Lake Palcacocha was reinforced with an artificial dam and more recently lake level monitoring and control has been conducted. In Chicón extensive retention measurements were mainly built in the headwaters (Ocururuyoc Pampa), halfway between the glacial lake and the settlements (Flores Moreno 2016). Nevertheless, the risk of GLOFs still remains in both areas.

Typical for the Peruvian Andes is a seasonal climate with a wet season between October and April and a dry season between May and September. During the dry season, people in these catchments depend more strongly on glacial melt water (Mark and McKenzie 2007), which feeds the rivers that essentially support agricultural activities in the catchments and also contributes to water springs (Somers et al. 2018) that are important sources for domestic demand of people in the rural areas.

Huaraz, as the capital of the Region of Ancash and the major city in the area hosts a variety of industries (including mining) and companies for food, construction, housing etc. It is also the center of business, commerce, finances and tourism. In Quillcay traditional small-scale agriculture, spread over elevations between 3000 and 4000 m asl. dominates, and traditional crops like potatoes, corn and grains are produced for self-subsistence and the local markets (Gurgiser et al. 2016). The money earned is used to buy food products that cannot be cultivated in this environment such as rice, sugar and different types of vegetables and other consumer items. Livestock serves as a complementary reserve fund to reduce uncertainties related to varying agricultural crop production (e.g. due to weather patterns).

The economy of Chicón is mainly based on agriculture (cultivation of maize, potatoes, beans, and wheat), livestock (with a similar function as in Quillcay) and tourism (Flores Moreno 2016). Additionally, floriculture (flower farming), a water demanding crop, is a growing economy mostly for export into regional and national markets. Urubamba has been one of the main centers of exchange for goods and services of local communities and towns of different ecological zones in the Vilcanota-Urubamba valley.

Both areas are prominent tourist regions in Peru. Urubamba is a destination in the Sacred Valley of the Incas that connects Cusco and Machu Picchu whereas Huaraz is the hub for mountaineers and visitors of the Huascaran National Park (Carey 2012). Huaraz has the infrastructure to deal with a large number of visitors and therefore tourists do not have a pervasive impact on the local conditions. In Urubamba and Chicón the number of tourists relative to the local population is substantial and their impact on water demand is discernible while this effect is less important in Huaraz (Burga 2018).

## Methods and data

Our research sites in Peru present a multi-hazard environment where we focus on the two important and interplaying hazards related to water. First, we consider limited water availability and water scarcity, which we refer to here also as low-flow water hazard. Water scarcity is defined as the situation where insufficient water resources are available to satisfy long-term average requirements (EU. 2007) or as overexploitation of water resources when demand for water is higher than water availability. It incorporates social factors (demand), and is thus not solely based on a natural process and hence not a conventional hazard (Van Loon and Van Lanen 2013). For our study region, glacial melt water is an important variable in future water scarcity; and we therefore focus on the future changes in glacial melt water content based on two greenhouse gas emission scenarios, i.e. RCP2.6 and RCP8.5, which represent a lower and upper-end range, respectively, of likely future warming. To analyze the meltwater content we therefore use the glacial extent. Several studies have assessed the potential impact of glacier retreat in the Peruvian Andes. In this study we use results by Schauwecker et al. (2017), where they used climate variables to estimate the rise of the freezing level height and used it as an indicator of future glacier extents, showing that by the end of twenty-first century in a RCP8.5 scenario only 1% (3%) of the current glacier extent will remain in the Cordillera Blanca (Vilcanota). Other studies indicated that climate change is likely to lead to changes in runoff seasonality related to more direct precipitation runoff and reduced melt due to reduced glacial area (Juen et al. 2007; Huss and Hock 2018).

Second, we analyze GLOFs as an important high-flow hazard, which in the Peruvian Andes is known for devastating disasters destroying villages and claiming several thousand lives. We do not analyze other high flow hazards, such as floods, rain storms etc. although they could play a role in the area as well (Baraer et al. 2012). The aforementioned GLOF from Lake Palcacocha in 1941 was historically the most severe GLOF disaster in Peru (Carey 2010), and probably worldwide in terms of casualties. In 2010, a GLOF from Lake 513 affected the city of Carhuaz and several downstream communities (Carey et al. 2012; Schneider et al. 2014). GLOFs are in fact a notorious hazard for many Andean valleys in Peru, and climate change is an important driver because increasing temperatures cause glaciers to melt and lakes to form, some of which are prone to outburst floods (Harrison et al. 2018). GLOFs can produce peak discharge up to tens of thousands of m^3^/s (Walder and Costa 1996; Richardson and Reynolds 2000; Hubbard et al. 2005; Carrivick and Tweed 2016), and thus represent the extremes of high-flow water hazards. GLOFs are representing an ever-looming risk as their outburst can have multiple different triggers and limited time for warning remains (Hofflinger et al. 2019).

Multi-hazard assessments are challenging because different hazards can have coupled dynamics, relations, interactions, cascading effects etc., but may not be directly comparable as they require different metrics to describe their process characteristics (Kappes and Keiler 2012; Forzieri et al. 2016). Recent approaches distinguish between different types of interacting, interconnecting, compound and cascading risks, where multiple physical hazard process interact, overlay and exacerbate the impact (compound events) or physical processes are at the beginning of series connected impacts on human systems such as infrastructure (cascading risks) (Pescaroli and Alexander 2018). On the level of hazards other methods analyze how different types of hazards can interact (e.g. earthquakes, storms, landslides) using a matrix where all combinations of hazards can be systematically connected (Gill and Malamud 2017). Cascading processes and hazard interactions have high relevance for GLOFs because they are typically the results of a triggering process (e.g. a landslide or avalanche impacting the lake), dam failure processes and flow transformation and erosive processes along the flow trajectory (Worni et al. 2012; Westoby et al. 2014; Mergili et al. 2018). While we consider these complexities for the GLOF hazard assessment we strive for a better comparability between high-flow and low-flow water risks and their impact on the population. For reasons of comparability or interconnections of hazards methods have been presented to classify different physical hazard processes into a number of categories (e.g. low, medium, high) or normalize and weight their different units (Kappes and Keiler 2012).

We analyze water scarcity and GLOFs separately and then compare how many people, agricultural land and infrastructure are exposed to the two types of hazards. This approach basically follows widely established definitions of risk where risk is a function of hazard, exposure and vulnerability (IPCC 2014), and allows us to achieve comparability at a level directly relevant for people affected. We perform this approach for both study sites separately and put this assessment into a context of local perspectives and perceptions analyzed through surveys we conducted. In the following we describe the methods for the analysis of low-flow (water scarcity) and high-flow (GLOF) water hazards and local people’s perspectives.

### Assessment of water scarcity

Several studies in Peru have been carried out to assess both detailed hydrological processes (including glacier simulations) and climate change scenarios through the use of several tools and models with different complexity, with details being reported in these studies (Bury et al. 2011; Condom et al. 2012; Rabatel et al. 2013; Somers et al. 2016; Vuille et al. 2018).

**Fig. 2:**
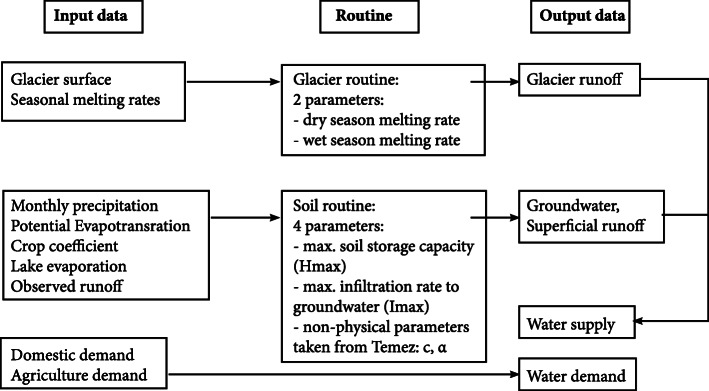
Scheme of the Temez modified hydrological model

In this study we use a lumped hydrological model with the ability to represent the most important findings from existing literature, differentiate glacier runoff from total runoff, and use limited input data which is a basic constraint in the study area. This simple model is based on a set of equations originally developed by (Témez 1977) which were further extended to incorporate additional important routines. The modified version simulates water supply from glaciers, groundwater and superficial runoff, while integrating water demand from agriculture and domestic use (for more details see Table 1). Glacier runoff is estimated as glacier surface times seasonal melting rates. The soil routine was calculated using the Temez equations in order to estimate groundwater and superficial runoff. The modified model comprises 6 parameters (2 seasonal melting rates for the glacier routine; 4 parameters for the soil routine), which were constrained using existing findings from literature (percentage of current glacier contribution, seasonal behavior, etc). Domestic water demand is based on l/capita/day based on assumptions by (Drenkhan et al. 2019) and adapted for the future based on population growth. Agricultural demand on the other hand was not changed for future scenarios, as it can be assumed that rising demand and improved infrastructure outbalance each other. Table 1 provides an overview of the input data and sources while Fig. 2 shows the scheme of the modified model.

**Table 1:** Input parameters and data for Quillcay and Chicon for the hydrological balance model based on Temez

	Input parameters/ data	Quillcay	Chicon
Supply	Precipitation	Monthly Precipitation data was collected from various weather stations between 1980 and 2015 in the area of Cordillera Blanca, interpolated and averaged based on the GRID cells for the Quillcay catchment area (Bárdossy and Pegram 2014)	Monthly Aggregated from daily measurements at SENAMHI weather station in PISAC from the years 1963 to 2014
	Daily glacier melting rates	Dry season: 4 mm/day Wet season: 10 mm/day (Kronenberg 2019)	Dry season: 4 mm/day Wet season: 8 mm/day (Kronenberg 2019)
	Potential evapotranspiration	estimated on the reference evapotranspiration (ETo) calculator based on the standard FAO Penman-Monteith equation (Allen et al. 1998), which uses maximum and minimum temperatures	estimated on the reference evapotranspiration (ETo) calculator based on the standard FAO Penman-Monteith equation (Allen et al. 1998), which uses maximum and minimum temperatures
	Temperature	Temperature was obtained from the ONERN and SENAMHI weather stations in Huaraz at 3200 m a.s.l. for the periods 1950 to 1954, 1965 to 1970 and 1978 to 1995 (PROFODUA 2008)	Monthly Min./Max. aggregated from daily measurements at SENAMHI weather station in PISAC from the years 1997 to 2014
	Evaporation	Monthly average using evaporation rates from Conococha lake at 4020 m asl (local report/source, not published)	Monthly average measured at Sibinacocha 4800 m asl 1981 to 1996 (EGEMSA)
	Runoff (used for calibration)	monthly average between 1953 to 1999 (ANA: AEGL)	few point measurements (Estudio hidrológico Chicón, Municipalidad provincial de Urubamba, 2012)
Demand	Domestic	120 l/capita/day with water leakages in the order of 45% (97 l/capita/day) (Drenkhan et al. 2019) Scenarios: population numbers based on INEI 2017 and growth rate of the region of Ancash	120 l/capita/day with water leakages in the order of 45% (97 l/capita/day) (Drenkhan et al. 2019) Scenarios: population numbers based on INEI 2017 and growth rate of the region
	Agriculture	based on the crop requirement of the existing crops (PROFODUA 2008) including net irrigation requirement, crop requirement, effective precipitation, planted area, ETo and the crop coefficient (Kc) according to FAO (Allen et al. 1998) and growth stages of the different crops	1 l/s/ha according to Diagnosis and Water Resources Management Plan of the Vilcanota Urubamba Sub-basin (ANA 2010)

Few studies, with different periods and robustness of modelling, have been carried out to estimate melting rates in the tropics (Wagnon et al. 1999; Favier et al. 2004; Winkler et al. 2009; Sicart et al. 2011; Gurgiser et al. 2013). The different authors identified different peculiarities for their investigation sites: distinct melting rates, with responsible energy fluxes and governing processes. Kronenberg et al. (2016) concluded that in the Cordillera Vilcanota changes in atmospheric moisture content are likely to lead to an important increase in melt energy. Sicart et al. (2011) analyzed the spatial and temporal variations in the mass balance of Zongo Glacier (Bolivia) showing that cloud cover and precipitation are responsible for the seasonal changes in melt energy. Melting rates on tropical glaciers depend more on the particular and local energy balance, where humidity plays an important role. In that sense, we used melting rates as model parameters to estimate glacier contribution as expected from existing literature.

The aim of the hydrological simulation is to assess possible changes in seasonal water availability from changes in glacier contribution due to climate change, and considering existing water demand. Therefore, the hydrological simulations were carried out in a monthly time step, setting up two periods: current situation (multi-annual average from 1980 to 2015) and climate change scenarios for the end of the twenty-first century. Note that our simulations are not transient but instead are based on average monthly values from the respective times periods (1980–2015, and 2071 to 2100).

For calibration, we used an objective function that minimizes the difference between measured and simulated runoff, taking into account the seasonal variability and changing iteratively the model parameters in order to represent the seasonal variation and a good fit with findings from existing literature in particular the percentage of glacier contribution to total runoff. A challenge for hydrological modeling and estimates in our case study regions (and in general for much of the Andes) are missing runoff observations. This is especially true for the Chicón catchments where very sparse runoff measurements were available, which induces a certain degree of uncertainty. As a result, for the Chicón catchment spare runoff measurements were used mainly to provide the order of magnitude for simulations.

For the climate change scenario, we do not consider changes in future precipitation, mainly because for the Andes of Peru climate models show a high variability in projections of rainfall change in the future, including both increase and decrease which eventually results in high uncertainty (Neukom et al. 2015; Otto and Gibbons 2017; Segura et al. 2019). Glacier area changes for the end of the century are based on Schauwecker et al. (2017), distinguishing between the effects of a low-emission (RCP2.6) and a high-emission (RCP8.5) scenario. Other model parameters like precipitation, temperature, evaporation, evapotranspiration and melting rates remain constant. Therefore, the model enables us to analyze the change of glacier melt water contribution over time and resulting future water availability changes.

### Assessment of GLOFs

In order to predict the area that is potentially inundated by a GLOF we use the numerical model RAMMS (Rapid Mass Movements Simulation) for debris flows (http://ramms.slf.ch/ramms/), which has been successfully applied for GLOFs by Schneider et al. (2014) and Frey et al. (2016). RAMMS is a 2D model using a Voellmy approach incorporating a dry Coulomb friction μ and a turbulent friction ξ (Bartelt et al. 1999) to simulate mass flow processes.

For the assessment of the hazard of GLOFs for Quillcay Frey et al. (2018) presented a hazard and evacuation map for the city of Huaraz. The assessment is based on a multi-source scenario-based hazard assessment approach for the three critical lakes in the Quillcay: Palcacocha (with a volume of 17 × 10^6^ m^3^), Tullparaju (12 × 10^6^ m^3^), and Cuchillacocha (2 × 10^6^ m^3^). Each scenario includes the full process cascade from an impacting avalanche, displacement wave in the lake, and overtopping of the dam, to the outburst flood to simulate the different magnitudes with related probabilities of involved cascading mass movement processes. We adopt here the Frey et al. (2018) approach, and assign qualitative probabilities of occurrence (high, medium, low) to the GLOF magnitudes of the three different scenarios (small, medium, large, respectively). In order to simulate the GLOF scenarios RAMMS was used to model possible avalanches impacting the glacier lakes, followed by empirical estimations of wave generation and dam overtopping (Heller et al. 2009) and eventually again using RAMMS to simulate the GLOF downstream flow path propagation and extent of flooding down in Huaraz. Results of maximum flow height maps obtained by RAMMS simulations were then converted into intensity maps and corresponding hazard levels according to national and international standards (Raetzo et al. 2002; Schneider et al. 2014; GAPHAZ 2017), and eventually combined into a GLOF hazard map for the entire Quillcay catchment, including the urban area of Huaraz (Frey et al. 2018). The simulation is based on a LIDAR digital elevation model (DEM) from 2010 with 5 m spatial resolution from the Peruvian Ministry of Environment (Ministerio del Ambiente, MINAM). The frictional parameters in RAMMS were set to μ = 0.08 and ξ = 500 m s^− 1^ in these steeper regions, and μ = 0.04 and ξ = 500 m s^− 1^ for flatter areas (cf. Schneider et al. 2014; Frey et al. 2018). Initial flow volumes for different scenarios vary between the three lakes but are between 0.15 × 10^6^ and 2 × 10^6^ m^3^.

The hazard map based on Frey et al. (2018) was then used to look at the elements at risk in the lower Quillcay catchment and Huaraz. Therefore, exposure of people and infrastructure was determined by overlaying the extent of the potential runout with urban areas, irrigation areas, and infrastructures such as roads, water channels for irrigation as well as water extraction stations to express mainly the risks for the water distribution and impacts for the local population. This data was provided by openstreetmap (Geofabrik GmbH 2018) and local contacts.

For Chicón the simulation of the GLOF process cascade was simplified without explicit modeling of GLOF trigger processes due to the variety of possible trigger process scenarios (such as ice break-off and avalanches, lake overflow due to heavy precipitation). Definition of GLOF magnitude scenarios were based on the 2010 event, local reports and potential water and sediment availability for potential future GLOFs. Water availability for a potential GLOF was based on lake volume estimates (Huggel et al. 2002; Muñoz et al. 2020), cf. Figure 1, while sediment availability was assessed during field work and using high-resolution satellite image analysis. The total volume of the 2010 outburst is estimated to be around 75.000 m^3^ (Silva et al. 2018), including both liquid and solid parts of the GLOF. Based on this analysis, we used this magnitude as an orientation for a small-scale scenario. The medium-scale scenario (total GLOF volume of 200.000 m^3^) is assumes a quasi-complete drainage of Lake Riticocha (110,000 m^3^ of water) and sediment erosion and entrainment on the same order of magnitude along the flow path. The large-scale scenario represents a worst case (500.000 m^3^ total volume) that may result from different physical processes and their interaction or accumulation, including full drainage of Lake Riticocha and massive entrainment of highly saturated sediment due to large antecedent rainfall as recently observed in other alpine environments (Walter et al. 2020) and deep erosion of thick sediment bodies (such as the debris fan at Ocururuyoc Pampa) which can result in multiplication of flow volumes as observed elsewhere (Huggel et al. 2012).

Topographic basis for the simulation is an artifact- and void-corrected Digital Elevation Model (DEM) from the global Shuttle Radar Topographic Mission (SRTM), acquired in 2000 upscaled from originally 30 m to 12.5 m spatial resolution (ASF 2015). The frictional parameters were defined based on several model runs and calibration, and set to μ = 0.06 and ξ = 500 m s^− 1^; these correspond to the slope of the flow and debris fan on which the city of Urubamba is built and Chicón’s gradual declining steepness. After the GLOF in 2010, disaster prevention measures have been implemented by the provincial municipality of Urubamba (Flores Moreno 2016). The most outstanding structural work is in Ocururuyoc Pampa. It consists of several defense lines, dams, sediment retention structures and a number of tubes for drainage under normal conditions as well as a concrete dam in a small canyon at the exit point of Ocururuyoc Pampa (Fig. 4). Field surveys, however, indicated potential structural instabilities of the dam in case of flooding (potential erosive undercutting at the lateral foundations). We therefore included scenarios and model runs both with and without the dam. In the absence of a detailed structural and hydraulic dam failure analysis scenarios without consideration of the dam mimic a situation of dam failure. For scenarios without dam failure, we included the structure of the dam in the DEM, thus simulating flood retention. Further downstream where it enters rural communities and urban areas the river is channelized, yet not with sufficient flow capacity in case of a GLOF. The channelized river structure was therefore not included in the DEM for simulations.

### Local perspective on risks

In 2018, we conducted interviews in Huaraz and in the villages surrounding the city in order to investigate how local residents and local authorities experts view low and high-flow water risks, specifically their view towards water, water related risks and adaptation strategies for these risks. The two interviewee groups were chosen beforehand to ensure differentiated perspectives on the subject. The approach for the interviews was based on snowball sampling (Patton 1990) to get access to the interviewees, followed by episodic, focused (Flick 2000) and expert interviews (Bogner et al. 2002; Bogner et al. 2009). Snowball sampling was chosen in regard to the purpose of the study. Due to long-standing research experience in the area, initial contacts were already established that further helped to broaden the group of interviewees. The sampling started with at least two different entrants to guarantee a variety of opinions. In the end, interviewees consisted of 11 people who live in the rural areas around Huaraz referred to as local people and 20 representatives from different governmental and non-governmental Peruvian institutions (international, national, regional and local) who work in the field of water and risk management, here referred to as technical experts. All interviews were transcribed, analyzed with the software MaxQDA and finally interpreted according to Mayring’s content analysis (Mayring 2007). Thereafter, the content was reduced and essential information was separated through a coding and category system to fit the research aim. All interviews were conducted in Spanish. While technically and scientifically we clearly distinguish between hazard and risk, local people and most of the technical experts interviewed did not make this distinction and used these two terms (in Spanish ‘peligro’ for hazard and ‘riesgo’ for risk) in an interchangeable way.

While interviews in Quillcay focus mainly on the comparison of perspectives of GLOF and water scarcity, interviews in Chicón are directed more to the narratives and meanings of water, which reflects the indigenous context of Chicón. The semi-structured interviews as well as participant observations in Chicón were conducted by Burga (2018) within the local villages in the Chicón valley and are based on an ethnographic approach to understand ways of living, meanings, and behavior (Bernard 2006) and also analyzed according to Mayring. In this respect, interview questions and observations are oriented towards the relation with Nevado Chicón, glacial lakes, and rivers and the link to the 2010 GLOF and the following risk management activities. Interviews were conducted in November 2017 to March 2018 with 48 people (14 women, 34 men) living in Chicón. Most interviews were carried out in Spanish and only a few cases were conducted in the local Quechua language with the help of a translator.

## Results

### Quillcay

**Fig. 3:**
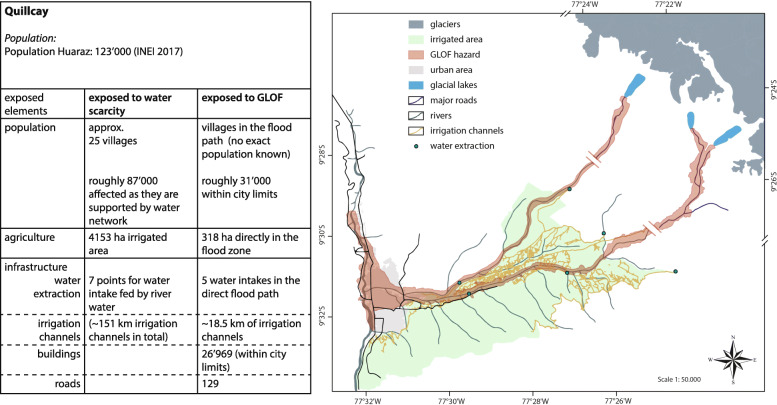
GLOF exposure for the Quillcay and city of Huaraz

Interviews in the Quillcay revealed that GLOFs and water scarcity (in the dry season) are the most mentioned hazards and therefore the hazards connected to water most relevant for people in the area. Local people as well as technical experts stated that GLOFs are considered as one of the major risks in the Quillcay, taking into account that the 1941 GLOF destroyed about one third of the former city of Huaraz. The GLOF modeling studies show the exposure according to the scenarios considered for Huaraz and surrounding areas (Fig. 3). Elements at risk include people, major infrastructure, including the city center of Huaraz, agricultural areas and water distribution in the Quillcay. In 2017, Huaraz counted approximately 123,000 inhabitants (INEI 2017) ten times as much as in 1941.

The two rivers Paria and Auqui are the veins for the water supply in the lower valley, where water for agricultural irrigation is extracted at seven points (water intakes). From each of those points starts a complex network of water channels that irrigate adjacent agricultural fields. Five of these are situated in the outflow path of potential GLOFs. Two near the river Paria fed by Lake Palcacocha and three in river Auqui fed by the lakes of Tullparaju and Cuchillacocha. A GLOF could therefore potentially influence almost the entire agricultural area of the nearby city of Huaraz, especially in times where harvest is dependent on river water and not rain fed or when carried debris could destroy fields nearby the river.

Concerning water availability, almost all residents report that there is not enough water in the dry season. According to them, river water has decreased over the last years, springs dried out and the amount of water is not enough anymore to irrigate the fields and supply drinking water at the same time. The opinion among technical experts is divided, where some already see water availability as a problem that could be exacerbated in the future and others, mainly experts from the National Institute of Civil Defense (INDECI) do not see a problem at all. Both technical experts and local people see drivers related to natural processes, climate change and glacier retreat as a cause. Additionally, both groups of interviewees mentioned social drivers such as population growth, agriculture and unsustainable domestic and irrigation water use as drivers of water scarcity. Further, local residents are also concerned about decreasing rainfall, drying of springs and drying mountains whereas technical experts consider other factors such as mining activities and the expansion of agriculture as well as changes in crops along with inefficient irrigation systems as a driving force of water scarcity. Some technical experts also reported that water shortages and rationing of water already happened. Local residents furthermore indicated that conflicts over drinking and irrigation water in the rural areas were emerging and people living close to the river were blamed of too much water usage.

While people’s views involve various drivers of potential water scarcity we took a further look on glacier retreat related water scarcity due to climate change. The results of the water balance model are shown in Table 2. The data rows (observation period, RCP2.6 and RCP8.5) reflect water supply minus water demand. Water demand only includes domestic and agricultural use, as these are the main users within the Quillcay. The water balance shows a distinctive smaller runoff in the months of the dry season (May to September) where runoff in the observational period goes down to below 3m^3^/s. During the wet season, precipitation is responsible for the much higher amounts of runoff (up to 12.0m^3^/s). The glacier area of Quillcay is 13% of the total catchment and glacier melt is an important part of the total runoff, especially valuable during the dry season when precipitation is lacking.

**Table 2:** Monthly water balance for the Quillcay and the Chicón catchment based on the historic simulated data and extended for the RCP scenario 2.6 and 8.5 for the end of the 21st centtury. Dry season approximately May to September, wet season approximately October to April

	Quillcay			Chicón		
	Observation period	RCP2.6	RCP8.5	Observation period	RCP2.6	RCP8.5
	m3/s	m3/s	m3/s	m3/s	m3/s	m3/s
January	8.8	7.8	7.2	1.08	1.01	0.97
February	10.6	9.6	9.0	1.15	1.08	1.04
March	12.0	11.1	10.5	0.96	0.89	0.85
April	7.9	6.8	6.2	0.46	0.39	0.35
**May**	**3.3**	**2.1**	**1.8**	**0.24**	**0.28**	**0.24**
**June**	**3.5**	**2.8**	**2.4**	**0.24**	**0.21**	**0.18**
**July**	**3.3**	**2.6**	**2.2**	**0.22**	**0.18**	**0.16**
**August**	**3.2**	**2.5**	**2.4**	**0.21**	**0.18**	**0.16**
**September**	**4.3**	**3.7**	**3.2**	**0.23**	**0.19**	**0.17**
October	7.3	7.8	7.1	0.37	0.30	0.26
November	9.0	8.0	7.3	0.46	0.39	0.35
December	9.1	8.1	7.5	0.63	0.56	0.52
Total	82.5	72.9	66.6	6.24	5.66	5.25
Average	6.9	6.1	5.8	0.52	0.47	0.44

While we assume in this simple water balance model that precipitation, evaporation remains constant for the future (end of twenty-first century), we reduce the glacier surface according to the two greenhouse gas emission pathways RCP2.6 and RCP8.5, representing low-emission and high-emission scenarios, respectively. The effect of differentiated glacier shrinkage on river runoff and water supply can be seen from Table 2 which is clearly discernible and relevant during the dry season: in the more optimistic RCP2.6 scenario the runoff goes down to 2.3m^3^/s and in the negative RCP8.5 scenario even down to 1.9m^3^/s. The model thus only calculates the effect of glacial retreat. However, only looking at the glacier retreat is not in balance with other climatic factors, which could accelerate glacial melt and therefore an even more pessimistic scenario might be possible. The retreat of glaciers has a major impact on a catchment like Quillcay where especially the runoff during the dry season is affected.

**Fig. 4:**
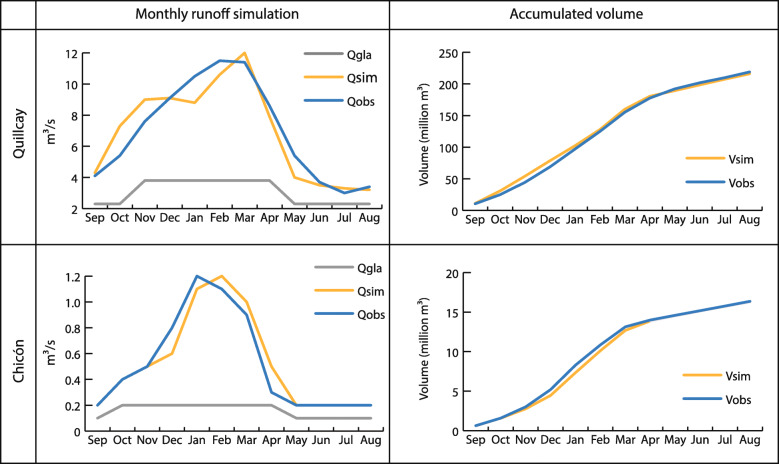
Hydrological calibration for Quillcay (upper) and Chicón (lower): [left] comparison between Qobs (observed streamflow), Qsim (simulated streamflow) and Qgla (glacier runoff); [right] comparison of Vobs (accumulated volume from observed total streamflow) and Vsim (accumulated volume from simulated streamflow)

Results from hydrological calibration for the current period (multiannual average from 1980 to 2015) are shown in Fig. 4 for both study sites, Quillcay and Chicón. The seasonal runoff pattern is reasonably well represented by the models as well as the accumulated water volume for the hydrological year. From them, glacier contribution was estimated as 67% (40%) during June, July and August (December, January and February) in the Quillcay catchment. In case of Chicón, glacier contribution was estimated as 49% (25%) during June, July and August (December, January and February). Both, seasonal pattern and glacier contribution are in line with results from literature (Condom et al. 2012).

Overall, the model does not show a water scarcity per se, where the demand outweighs the supply. Nonetheless, as mentioned above the availability of water during the dry season is considered as very limited, especially by local people, and generates conflicts, as e.g. between local water users. Furthermore, the model does not consider an environmental baseflow to sustain the environment. The environmental baseflow can be calculated in various ways and can therefore differ (Tharme 2003). Drenkhan et al. (2019) consider an environmental flow requirement of 5% of the average monthly discharge (without demand), as defined by the National Water Authority (ANA). Furthermore, changes in precipitation patterns, which are uncertain for the future (according to climate models) (Neukom et al. 2015) and not considered here, could greatly exacerbate problems of water availability and scarcity.

Some scholars argue that factors like population growth, water allocation practices and power relations are going to outweigh the climate change driven water supply (Buytaert and De Bievre 2012; Drenkhan et al. 2015; Jurt et al. 2015; Carey et al. 2017; Mark et al. 2017; Vuille et al. 2018). However, the interviewees rather focus on natural drivers of water scarcity when talking about the phenomenon. They seem to have a local view on the problem as they report the declining water resources on their direct environment (e.g. vanishing glaciers).

When talking about the most important risk, local residents responded mainly with water scarcity, technical experts on the other hand are concerned about GLOFs and water scarcity almost equally. However, some of the local people shared the opinion that both hazards are equally important and would not consider one over the other. When talking about water scarcity, interviewees often referred to “the water problem” in contrast to actually naming it a hazard or risk.

### Chicón

In Chicón, to understand the role of water and glaciers for the local people we first need to focus on the meaning and narratives of water, and do not explicitly compare different perspectives of locals or technical experts as done for Quillcay. Furthermore, while an in-depth description of the relation of local people to the *Apus*, the deities of the mountains, and its meanings is beyond the scope of this study, we acknowledge that the *Apus* have a higher power in the interpretation and meaning of water (cf. Vergara et al. 2007; Bolin 2009; Morrissey and Oliver-Smith 2013; Allison 2015; Burga 2018; Scoville-Simonds 2018). Water is seen as the element that structures the space in which land, people, and other elements of nature come together and interact (Gagné and Rasmussen 2016). Hence, how the water flows through the basin leads to distinct types of organization of the water sources where two spaces can be distinguished (Burga 2018). First, Nevado Chicón (including the lakes and the plain of Ocoruruyoq) (see Fig. 6) is the place where the water is born and therefore the primary provider of water. The *Apu* is a sacred entity that lives in the glacier and owns the water sources. Second, the basin is where the water flows. This area is where all the activities such as irrigation of agriculture, livestock herding, human consumption or others take place and contain the necessary infrastructure. Consequently, the recognition of the *Apu* and its protective entities is very powerful (Bolin 2009; Paerregaard 2013; Allison 2015; de la Cadena 2015; Burga 2018; Scoville-Simonds 2018) and should be given respect throughout different practices e.g. *pagos* (‘paying’, i.e. offer to the *Apus*) to ensure the access to water. Deglaciation is affecting the capacity of the glacier and the *Apus* to provide water. Indeed, climate change is widely identified throughout Chicón as a process causing changes (Cruz Rivera et al. 2017; Burga 2018).

The catchment of Chicón has a current glacier coverage of more than 6%. In general, the runoff in Chicón is low, barely exceeding 1.2m^3^/s even during the wet season (see Table 2). Runoff during the dry months is very low and goes down to only 0.2m^3^/s. The glacier melt water contribution is comparatively low but still has an important impact. The situation potentially worsens in the future, where the runoff in the high-emission scenario RCP8.5 is constantly below 1m^3^/s and goes down to 0.1 m^3^/s. The reduction in glacial melt water contribution would significantly decrease the water availability in the catchment.

Since we assume an average use of water during the year the water availability is not yet negative, which does also in Chicón not per se indicate conditions of water scarcity. Nevertheless, water scarcity is being recognized in the catchment and has mainly been attributed to the greater use of water for irrigation in the upper part of the local communities, mainly due to new economic activities like floriculture, leaving less water for the lower basin, where also the construction of condominiums and hotels adds to the competition over available water. Consequently, water distribution becomes the main cause of tensions and disagreements and poses a greater pressure on the water system and allocation (Cruz Rivera 2017; Burga 2018).

The 2010 GLOF originating from Lake Riticocha impacted the valley and the city of Urubamba. According to the Institute of National Civil Defense (INDECI 2010) the GLOF directly impacted 141 families and affected 481 more; 29 houses were destroyed and 76 damaged; 3 km of roads got destroyed and an additional of 3.5 km roads were affected; it left 6 bridges destroyed and affected 7 more. Furthermore, the GLOF affected 21 ha of agricultural land, destroyed 22 km of irrigation channels and 400 animal got hurt; it further affected the local water and energy supply. There have also been reports of a GLOF in 1942, which caused the death of 70 people, destroyed a number of houses and infrastructure such as irrigation channels (Flores Moreno 2016; Cruz Rivera 2017; Burga 2018).

Even though local governments have taken political decisions to protect the population against potential glacial hazards and disasters, including the installation of an early warning system, they systematically ignored the population and excluded it from decision-making processes. Hence, the aforementioned disaster prevention measures were not well received by the communities. The measures did not resonate with the peoples values and close connection to nature, as these structures are very unnatural (Flores Moreno 2016). Glaciers, glacial lakes or water do not mean the same for institutional and political actors and local people and social organizations. They all value these goods and places from different beliefs and social practices. While mining and hydroelectric companies view glaciers and water primarily as consumptive resources, local communities interpret these assets as preconditions for life. They fear losing these assets and resources. Thus, conflicts arise because local governments and institutions take decisions or seek to technically control the behavior of others, without proper processes of communication or public discussion. Generating mistrust and preventing cooperation and collective action, can increase vulnerability to climate change and the economic and political pressures of the context (Flores Moreno 2016; Burga 2018). However, even with the disorganization of institutions, these processes have also triggered dynamics of individual emancipation. The GLOF 2010 resulted in collective actions to protect the glacier and to preserve water resources. It led to a recognition of “new” places in the area of the lake Riticocha and Ocururuyoc Pampa, an understanding of new landscapes and new forms of social organization with new roles and responsibilities to face new contexts and new rules of coexistence and further increased the level of awareness towards water sources (Flores Moreno 2016; Burga 2018).

**Fig. 5:**
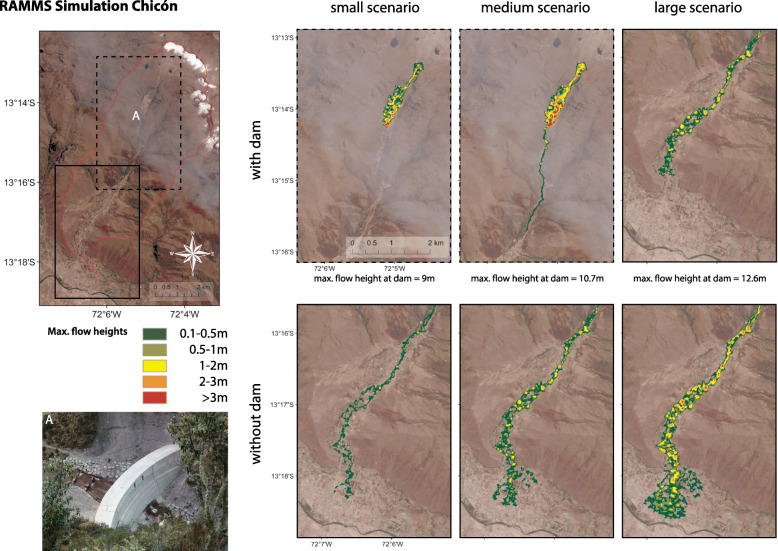
Outflow paths of potential GLOFs under different scenarios (volumes) modelled with RAMMS. Upper figure: runout without any measures in place. Lower figure: considers a 10 m high dam at the outlet of Ocururuyoc Pampa

Nevertheless, shrinking glaciers and growing lakes still present the danger of future GLOFs (Drenkhan et al. 2019). Due to the narrow topography of the Chicón valley, most of the population settles on the narrow valley bottom in close proximity to the Chicón river where also the agricultural land is located, which unfortunately coincides with the path of GLOFs. Figure 5 shows the GLOF hazard of the Chicón catchment (for different scenarios) modelled with RAMMS. It includes scenarios with and without the new dam that was built at the exit point of Ocururuyoc Pampa (Fig. 5). The dam is about ten meters high and has only a very narrow 0.9 m diameter water outlet. Capacity for material to be hold behind the dam is approximately 50.000m^3^ (Silva et al. 2018). Depending on the amount of material that would be released during a GLOF the danger of an outburst and destruction in Chicón still persists, which is why we also included model runs without the dam.

**Fig. 6:**
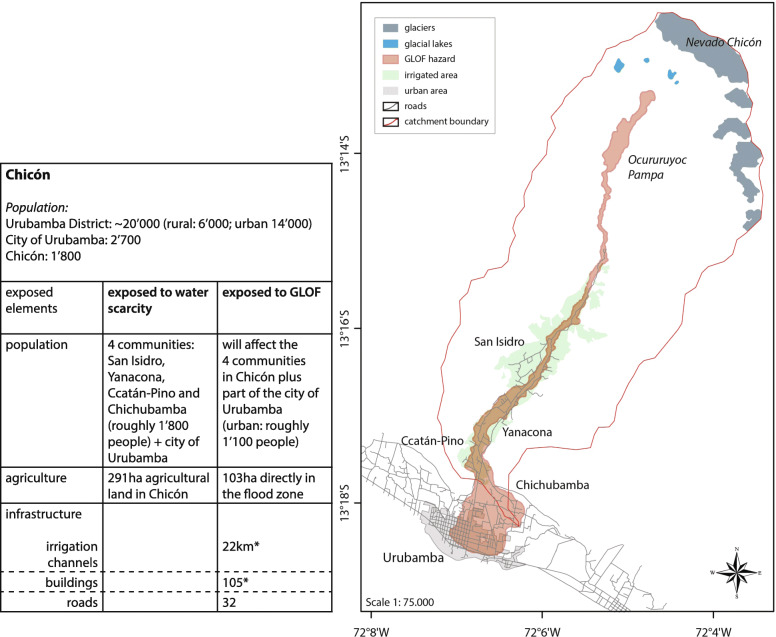
Exposure to GLOF hazards in Chicón and Urubamba (*) refers to the numbers of the 2010 GLOF (INDECI 2010)

In Fig. 6 elements at risk for the worst-case scenario of a GLOF are visualized. The GLOF flow path is in accordance with the large scenario modelled with RAMMS and considers flow heights > 0.1 m. Shown in the map are the irrigated area, roads and urban area. Elements of risk are listed, which gives a more detailed indication of exposed elements not just for GLOFs but also an estimation for water scarcity. Contrary to GLOFs no infrastructure is affected in relation with water scarcity problems, but due to the dependency of livelihoods on irrigated agricultural land water scarcity affects a larger amount of people (Cruz Rivera 2017).

## Discussion

Our two study sites Quillcay and Chicón show several similarities with respect to water resources and related risks. Both are glaciated catchments in the Peruvian Andes with a similar climate and water regime, significantly affected by effects of climate change and glacier shrinkage, which creates a multiple-hazard environment. Both Quillcay and Chicón are confronted with risks related to limited water availability (or water scarcity, depending on the perspective) and GLOFs. Both catchments are characterized by small-scale agriculture and an adjacent larger city that offers markets and a connection to the region. Nonetheless, a hazard or risk assessment that would comprehensively consider both types of water related risks is not very straightforward and offers multiple challenges. For instance, narratives of water, meanings of water and the role of the *Apus* play an important role in the perception of and discussion about water hazards and risks. In both catchments, there is a disparate distribution of the priorities between the management of hazards and risks. The priorities of state institutions lies in adverting of GLOF risks rather than giving priority to water scarcity problems. For the local populations livelihoods are at risk if there is not enough water available and therefore the feeling of losing water is much stronger. However, local people do not neglect risks from GLOFs and are well aware of the risk proposed by glacial lakes.

Risk management in Peru is part of the legislation since 2011 with Law No. 29664 and the creation of the National System for Disaster Risk Management (SINAGERD), as approved in the National Policy on Disaster Risk Management in 2012 and the National Plan for Disaster Risk Management (PLANAGERD) in 2014. Nonetheless, translating the legal framework into risk management practice has not yet been achieved at a greater scale (Muñoz et al. 2016). Furthermore, a recurrent issue also seen in our study sites is the exclusion or marginalization of the local population from decision-making processes by local or regional governments and state institutions when decisions on natural resources are taken or when measures are taken to protect the population against potential glacial hazards and results in local resistance to risk management actions and measures because they are perceived as being imposed from the outside. The problem is that this process not only distances democratization and can lead to injustice, but also, by generating mistrust and preventing cooperation and collective action, it can increase vulnerability to climate change and the economic and political pressures.

Uncertainties arise in the qualitative assessment of local perspectives. Not all people have experienced natural hazards before nor do they know the exact classification of them. Hence, hazards, risks and dangers in general can be misinterpreted and experiences towards certain problems exacerbated. Water scarcity is perceived differently and not all water sources are connected to glacial retreat (e.g. drying of springs). Gurgiser et al. (2016) noticed in their study that locals perceive shifts in precipitation patterns that are not obvious in their precipitation analysis but shows for example a shift in onset dates of the wet season. Interviewees often referred to a “water problem” in disasters (Flores Moreno 2016). Therefore, locals’ perspectives have to be put in the respective context but should be taken seriously, as conflicts arise because some actors take arbitrary decisions or seek to technically control the behavior of others (Gagné et al. 2014), outside of processes of communication or public discussion and hence disrespect indigenous and community water rights (Lynch 2012). Muñoz et al. (2016) who studied a GLOF in 2010 that impacted the Chucchún catchment and the city of Carhuaz (close to Quillcay and Huaraz) found that the Peruvian state has limited capabilities to implement proper risk management due to the lack of knowledge that each authority has on current regulations, their roles and responsibilities. Further, Carey et al. (2012) emphasized missing involvement of population risk management processes often general while not classifying it. Hence, water scarcity could be misinterpreted, have multiple facets or be generalized. Reported water shortages for drinking water in Quillcay for example can also evolve due to the minimized capability of water treatment due to high sediment influx. Further, findings suggest that people’s perceptions of water related risks do not entirely correspond to scientific observations, which does not neglect them but raises the concern about other issues such as water competition between different users, water allocation etc. Limits to the comparison of two sites in our study is given due to the different scales of the catchments and methods used, e.g. other than for Chicón, GLOF modelling in Quillcay specifically considers impacts from avalanches and landslides as GLOF triggers, or the approaches for the interview with local people in the two catchments were different.

**Fig. 7:**
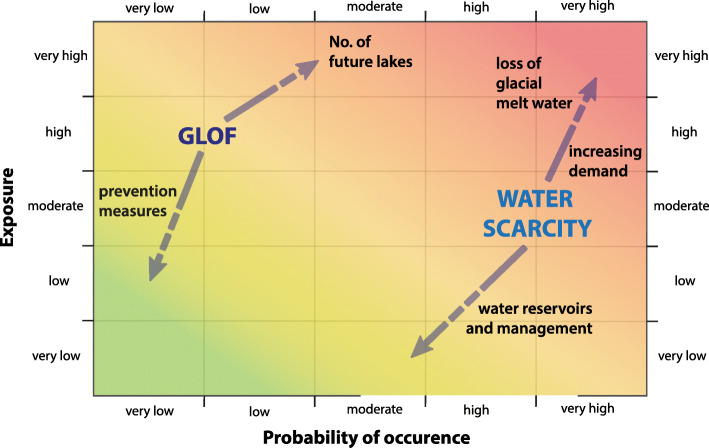
Matrix that compares probability of occurrence and exposure for GLOF and water scarcity. Arrows indicate future tendencies due to named processes

The past few years have seen important progress in multi-hazard and multi-risk research and a series of new or adapted approaches have been proposed (Kappes and Keiler 2012; Mignan et al. 2014; Scolobig et al. 2017; Schauwecker et al. 2019; Tilloy et al. 2019) of which several ones investigate how compound, cascading and interrelated hazards and risks can conceptually be assessed. Our issues here, seen with the two case studies in Quillcay and Chicón, i.e. how two types of risks can be quantitatively compared and set into perspectives of different local actors, is a bit less in the focus of the above studies. For this purpose we use a matrix (Fig. 7), similar to the one by Komendantova et al. (2014) to compare the two different hazard types found in our research area and to more systematically understand their differences and the effects of different risk management actions. It reflects the current situation but also tries to show future tendencies according to possible future developments.

**Fig. 8:**
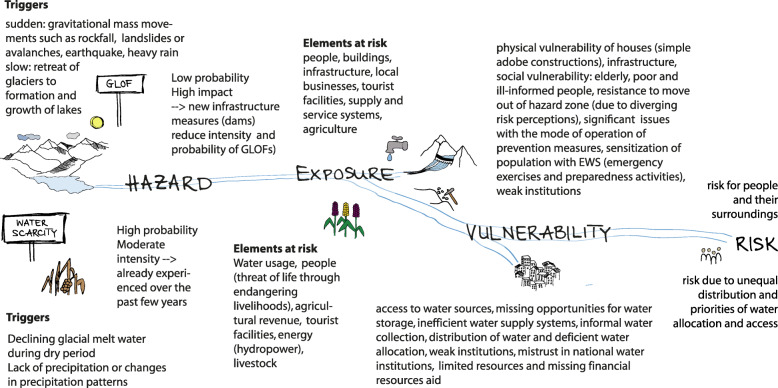
Scheme of trigger, hazard, exposure, vulnerability and resulting risks for GLOF and water scarcity; These processes are connected on different levels such as water allocation (periodically), water reservoirs and management, damage of infrastructure by GLOFs influences water availability for the period of destruction, glacial retreat and livelihoods

The two studied hazards water scarcity and GLOFs differ in their nature, intensity and return period. Water scarcity is a slow-onset process that is generated through climate change impacts due to receding glaciers and changes in precipitation patterns as well as through the unsustainable use of water. GLOFs on the other hand are a sudden-onset process that cannot only be traced back to climate change but whose possibility of occurrence is increased due to the melting of glaciers and the formation or growth of lakes (Harrison et al. 2018). The magnitude of the two hazards are measured in different ways with different units of reference, for example inundation depth for GLOFs and lack of water for water scarcity (e.g. in m^3^/s or days without water) (Carpignano et al. 2009). Figure 8 compares the triggers, hazard, exposure, vulnerability and risk of the two processes and highlights on which level they interact. To make the two hazards more comparable we modified the matrix that generally compares probability and intensity and plot probability of occurrence and exposure instead. We use exposure because intensity is not trivial to compare for the two types of water related hazards and because exposure allows us to say something in terms of risks. Exposure is space related and involves roughly the same people and infrastructure that are influenced by both hazards and is thus more comparable in our case. The matrix does not explicitly consider vulnerability of exposed people and assets, which would be important to more fully capture current and future risks but is difficult to determine in space and time. The matrix hence can offer a comparison of different hazards in terms of possible consequences with respect to loss of life or other possible impacts such as people, buildings, and infrastructure (cf. Kappes and Keiler 2012).

The definition of GLOF and water scarcity in the matrix (Fig. 7) reflects that the probability of a GLOF in our study areas is rather low. In Quillcay, for instance, GLOFs from Lake Palcacocha have been recorded in 1941 and 2003 but the lake has substantially grown since then. With water volumes of up to 10^6^m^3^ GLOFs from Lake Palcaocha have a high destructive power, as the 1941 example shows (Vilímek et al. 2005). Therefore, almost anything located within the flood path is directly exposed to this hazard. On the other hand, water scarcity has a high probability of occurrence as compared to GLOFs. It has been repeatedly reported during the last years (pers. communication local authorities in Huaraz, interviews and local reports) and can potentially occur on a yearly basis during the dry season. The number of exposed elements in our two case studies differ. But if we consider that Chicón is a much smaller catchment than Quillcay the relative number of exposed elements is similar. Therefore, we just use one matrix here to compare the two hazards. The exposure to GLOF is relatively high as more or less everything that ends up in an outflow path could be destroyed or disrupted due to the high amount of water, velocity and pressure of the flow. People die, agricultural land gets flooded or washed away, infrastructure and houses damaged or destroyed. Water scarcity on the other hand can involve the population of the entire catchment or even further downstream, hence the whole population could potentially be affected if not enough fresh water to fulfill the needs for human consumption is available. Agriculture is one of the main elements affected, as crops can get lost if insufficient water is available. Infrastructure or buildings are hardly directly affected but low-flow conditions are likely to have a negative effect on generation of hydropower or the amount of water used for other industries such as mining could be limited, further it will have economic impacts and damages, for instance in the form of loss of income due to crop loss. With all the effects mentioned it has a great influence of the livelihoods of people. Overall, for our case studies, we expect the exposure to water scarcity to be moderate.

We also include future natural and socio-economic developments and risk management actions and their effects in the matrix. The hazard and risks of GLOFs could be significantly reduced by prevention measures, such as lake lowering, flood retention measures, artificial dams or early warning systems. Nonetheless, the number of lakes is supposed to rise in the future with continuous glacier melt (Colonia et al. 2017), and lake levels are higher than ever (Lake Palcacocha) (Frey et al. 2018), which raises the probability of occurrence and intensity of a GLOF. Water scarcity on the other hand is not simple to manage under the prospects of glacial melt and uncertainties in precipitation. While we focused here on future water quantity, water quality can additionally exacerbate the problem. In the Quillcay, the tributary Auqui is known for its acidity due to high loads of aluminum, iron, manganese and lead, and glacier retreat plays a role in this process as additional bedrock with acid rich minerals become exposed (Fortner et al. 2011; Guittard et al. 2020). In addition, washouts as well as wastewater from mines can contaminate the river water further (Guittard et al. 2017). Furthermore, the increasing water demand puts pressure on the water availability. Therefore, the intensity of water scarcity could be higher in the future. Water management including better irrigation systems, reduction of water loss, regulated allocation, control of water quality etc. could reduce the intensity and probability of water scarcity, thus pointing to the potential and need of adaptation measures to be developed.

Further considerations that are not depicted in the matrix are interconnections of GLOFs with impacts on water availability and scarcity, and other types of services (Fig. 8). They can cause a disruption of public services, damage infrastructure such as roads, bridges, or affect the local and regional water system, irrigation canals and disrupt fresh water supply. By erosion and debris deposition they harm agricultural landholdings, agroforestry reserves and livestock, which are vital for agricultural productivity and maintenance of livelihoods (Carey et al. 2012). Thus, hazard relations and interactions may have unexpected effects and threats that are not captured by means of separate single-hazard analyses. Matrices such as proposed by Gill and Malamud (2017), or cascading process paths by Schauwecker et al. (2019) support the identification of potential hazard and risk interactions.

Overall, our two case studies reveal the diversity and complexity of issues that shape water related risks in Andean catchments. While we have seen that risks are very place-based our analysis also underlines the importance of risk assessment being more comprehensive and considerate of diversity in perspectives and environmental and socio-cultural context. Disasters are not only natural, but involve recognition of other forms of believes, economic processes of utilization of goods and political decisions about the distribution of such goods. In this process, it is important to move beyond informing local people to establish a dialogue, communicate and respect their way of life and involve them in such processes. Close engagement with local perspectives thus reassesses common frameworks (Jurt et al. 2015). This is necessary in order for a successful climate change adaptation and disaster risk management. The situation of water scarcity presents a challenge for integrated water resources management (IWRM) to meet (i) population needs and activities, (ii) reducing poverty and food insecurity, and (iii) contributing to environmental sustainability. In Peru, as well as other regions, it is necessary to reform the water resources management integrating the public and private sectors and civil society to achieve sustainability in the availability of the resource (quantity and quality) for present and future generations where water availability is often a matter of institutional arrangements (Rasmussen 2015). The current reality in Peru and many other countries is, however, that low and high-flow water hazards and related issues are handled separately, often by different institutions with limited or no coordination, even though on paper more integrated approaches are often promoted. Furthermore, our case studies also underline the particular importance of water demand, as a main driver of current and future water availability and potential scarcity. This corroborates findings identified in many other settings (Carey et al. 2017); and research is now increasingly integrating water demand into models and future projections (Brunner et al. 2019; Immerzeel et al. 2020).

## Conclusion

The two case studies presented here from the Andes in Peru show that both water scarcity (low-flow water risk) and GLOFs (high-flow water risks) could have important impacts on local population, infrastructure and economic activities. In particular, when it comes to design and implementation of adaptation or risk reduction measures the cases demonstrate the importance of integratively considering different water related risks. However, there is still limited research on combined comprehensive approaches, and likewise, the institutional structures and responsibilities in Peru and many other countries are a barrier to more integrative approaches. In the case of Chicón substantial efforts and financial investments have been made to reduce the risk of GLOFs. Nonetheless, missing acceptance on the local level and different meanings of water challenge the success of measures in place. Modernization and associated economic transformations exacerbated by globalization are important drivers of social and economic processes and undermine social identities in these localities. In the Andean culture and elsewhere water is the dynamic principle that explains movement, circulation and forces of change. It is critical to consider that forms of beliefs and traditions other than those of experts or scientists influence the decisions of local people and have an effect on vulnerability, perceptions of risk and thus implementation of adaptation and risk reduction measures.

In glacierized catchments where glacier melt is an important source of water in the dry season climate change and glacier shrinkage combine with rising water demands to exacerbate water scarcity in the future as we have shown using a relatively simple water balance model. This type of simple but rather robust models may be a reasonable choice in conditions of many mountain regions worldwide where availability and quality of data are a great challenge, in particular long-term river runoff measurements.

Comprehensive water risk studies therefore do not only need to consider low and high-flow water risks but also aspects of water supply and demand, as critical drivers of potential or effective water scarcity, such as discrepancies in water usage, which are often linked to issues of access to water, equitable distribution of water, power relations, or economic deprivation. Given these perspectives, it is clear that strong interdisciplinary efforts are needed to address the challenge. Moreover, to put scientific water risk studies into value for affected communities and institutions approaches of co-production of knowledge should be pursued where initial objectives are defined and problems framed together with stakeholders and results feed into adaptation and risk management measures.

## Data Availability

Data generated or analysed during this study are included in this published article or is cited at the appropriate space. As main sources of data can be highlighted the following citied articles: Burga MD (2018) The meanings of water: changing landscapes and water management in a sub-basin from the Southern Peruvian Andes. University of Zurich. Master Thesis. Flores Moreno A (2016) La sociedad puesta a prueba por el cambio climático: Una apreciación crítica de los actores principales de Canchis y Urubamba (Cusco), y su relevancia para la intervención del proyecto Glaciares+. 115. Frey H, Huggel C, Chisolm RE, et al. (2018) Multi-Source Glacial Lake Outburst Flood Hazard Assessment and Mapping for Huaraz, Cordillera Blanca, Peru. Front Earth Sci 6:210. doi: 10.3389/feart.2018.00210 Schauwecker S, Rohrer M, Huggel C, et al. (2017) The freezing level in the tropical Andes, Peru: An indicator for present and future glacier extents. J Geophys Res 122:5172–5189. doi: 10.1002/2016JD025943 Further data on the interviews in Quillcay can be found in: Thuer A (2018): Perceptions of low and high flow water risks in the Cordillera Blanca, Peru. University of Zurich. Master Thesis. OpenStreetMap information was taken from https://www.geofabrik.de/
